# Genomic analysis and mechanisms exploration of a stress tolerance and high-yield pullulan producing strain

**DOI:** 10.3389/fgene.2024.1469600

**Published:** 2024-09-20

**Authors:** Jing Yang, Ning Sun, Wenru Wang, Ruihua Zhang, Siqi Sun, Biqi Li, Yue Shi, Junfeng Zeng, Shulei Jia

**Affiliations:** ^1^ School of Basic Medicine, Shanxi Medical University, Taiyuan, Shanxi, China; ^2^ Department of Psychiatry, First Hospital of Shanxi Medical University, Taiyuan, Shanxi, China; ^3^ First clinical medical college, Shanxi Medical University, Taiyuan, Shanxi, China; ^4^ Department of Cardiology, Peking Union Medical College Hospital, Peking Union Medical College & Chinese Academy of Medical Sciences, Beijing, China; ^5^ School of Basic Medical Sciences, Tianjin Medical University, Tianjin, China

**Keywords:** pullulan and biomedicine, secondary metabolites, adaptive evolution, comparative genomics, Aureobasidium spp

## Abstract

Pullulan is a kind of natural polymer, which is widely used in medicine and food because of its solubility, plasticity, edible, non-toxicity and good biocompatibility. It is of great significance to improve the yield of pullulan by genetic modification of microorganisms. It was previously reported that *Aureobasidium melanogenum* TN3-1 isolated from honey-comb could produce high-yield of pullulan, but the molecular mechanisms of its production of pullulan had not been completely solved. In this study, the reported strains of *Aureobasidium* spp. were further compared and analyzed at genome level. It was found that genome duplication and genome genetic variations might be the crucial factors for the high yield of pullulan and stress resistance. This particular phenotype may be the result of adaptive evolution, which can adapt to its environment through genetic variation and adaptive selection. In addition, the TN3-1 strain has a large genome, and the special regulatory sequences of its specific genes and promoters may ensure a unique characteristics. This study is a supplement of the previous studies, and provides basic data for the research of microbial genome modification in food and healthcare applications.

## 1 Introduction

Pullulan obtained by microbial fermentation is a kind of natural macromolecular polysaccharide, which has many biological activities and healthcare effects. It is a kind of α-glucan with the α-(1,6) and α-(1,4) glucoside linkage alternating. It has been widely used in medicine, health food and other fields, and has many effects such as immune enhancement, anti-tumor, anti-aging, and blood lipid lowering (Tiwari et al., 2019). For example, it can be used in the treatment of arthritis, inflammatory bowel disease and cancer. It can also be used as an auxiliary carrier of drugs to enhance the stability and bio-availability of drugs for the treatment of central nervous system diseases. Studies have shown that lulan nanoparticles can significantly reduce the expression level of oxidative stress markers in the hippocampus of mice models of neurodegenerative diseases, which can significantly improve the learning and memory ability of mice models of neurodegenerative diseases, and improve motor dysfunction (Wang et al., 2022). Microbial polysaccharide capsules, mainly pullulan, have also gradually emerged in the field of biomedicine. Traditional capsules are made of gelatin, glycerin and water, but gelatin is not suitable for many types of drugs due to its shortcomings such as water loss hardening, water softening and cross-linking curing (Dong et al., 2008). The oxygen permeability of pullulan is 1/300 of hydroxypropyl methyl cellulose capsules and 1/8 of gelatin capsules, which can well protect the capsule contents from oxidation (Singh et al., 2023). In addition, pullulan also has a good regulatory effect on the human immune system, which can enhance the immune function of the body, and improve the body’s resistance. *Aureobasidium* spp (Taxonomy ID: 5579) is a genus of ascomycete fungi in the family Saccotheciaceae. They are well known for their great biotechnology potentials (Jia et al., 2020; Chen L. et al., 2020; Chen TJ. et al., 2020; Chen et al., 2018; Zhang et al., 2018). The pullulan found so far was mainly synthesized by the *A. pullulans* and *Aureobasidium melanogenum* lineages (Chen et al., 2018). As a promising microbial polysaccharide, how to increase the yield of pullulan by screening or improving microorganisms is an urgent problem to be solved. Studies showed that *A. melanogenum* TN3-1 isolated from honey-comb can produce high levels of pullulan and can withstand high osmotic environment (Chen L. et al., 2020; Chen TJ. et al., 2020). Compared with other strains of *Aureobasidium* spp., this strain has the stress tolerance and potential research values (Chen L. et al., 2020; Xue et al., 2019). Isolates of *A. melanogenum* TN3-1 from hyperosmolar honey was able to produce high levels of pullulans from high concentrations of glucose due to hyperosmolar tolerance, while other strains such as *A. melanogenum* P16 and *A. melanogenum* CBS105.22 were not able to produce high levels of pullulans from high concentrations of glucose due to hyperosmolar intolerance. Especially, strain CBS105.22, isolating from granuloma, could not be able to produce any pullulan (Chen L. et al., 2020; Chen TJ. et al., 2020; Chen et al., 2018; Zhang et al., 2018; Xue et al., 2019). These different phenotypic differences may be closely related to their genomic variations during evolution. Although the differences and molecular mechanisms of high pullulan producing strains from *Aureobasidium* spp. have been analyzed at genome level (Jia et al., 2023; Gostinčar et al., 2014), the mechanisms of high pullulan producing strains is still not fully resolved. Therefore, on basis of the available studies, we conducted a genomic comparison of *Aureobasidium* strains from different sources, and further analyzed the gene regulation and related signaling pathways from a new perspective, so as to provide supplementary for improving the pullulan production, and increase its applications in capsules, medicine or disease therapy.

## 2 Materials and methods

### 2.1 Genome download and collation

Based on the existing literature (Chen L. et al., 2020; Chen TJ. et al., 2020; Chen et al., 2018; Zhang et al., 2018; Xue et al., 2019; Jia et al., 2023; Gostinčar et al., 2014), we downloaded the reported genomes of *Aureobasidium* spp. from the NCBI (https://www.ncbi.nlm.nih.gov/assembly/), and organized the phenotypic information of them. In addition, we downloaded other fungi genomes of Ascomycetes for comparison, including *Aspergillus niger*, *Saccharomyces cerevisiae*, *Yarrowia lipolytica*, *Schizosaccharomyces pombe* and so on. All the data were summarized and collated for further genomic analysis.

### 2.2 Gene annotation and functional analysis

Sequence alignment was conducted between predicted genes and functional databases (BLASTP, E-value ≤ 1e-5), and the results with matching similarity ≥40% and coverage ≥40% were selected for annotation. The general functional databases annotated mainly included Swiss-Prot/TrEMBL, CAZy, GO, TCDB, KEGG and the NCBI non-redundant (NR) database. All known protein domains were searched by Pfam, ProDom, SMART database and InterProScan v78.0. Secondary metabolites biosynthesis gene clusters were annotated through antiSMASH v7.0 (https://antismash.secondarymetabolites.org/).

### 2.3 Gene alignment and genomic collinearity analysis

The α-1,3-glucan synthases (*Am*Ags2) were downloaded from NCBI according to the previous studies (Chen TJ. et al., 2020; Chen et al., 2018; Jia et al., 2023). Protein structure and function analysis was conducted through the NCBI-BLAST online website (https://www.ebi.ac.uk/Tools/sss/ncbiblast/), and the DeepTMHMM v.1.0.39 (https://dtu.biolib.com/DeepTMHMM) was used to conduct membrane structure domain analysis. The collinearity analysis of *Aureobasidium* spp. was conducted through MCScanX toolkit (https://github.com/wyp1125/MCScanX) (Wang et al., 2024).

### 2.4 3D structure prediction of the α-1,3-glucan synthase

We submitted the α-1,3 glucan synthase from P16, TN3-1, CBS105.22, and the type strain CBS10374 to AlphaFold Server 3.0 (https://alphafoldserver.com/) (Abramson et al., 2024). The protein three-dimensional structures of crucial genes involved in the synthesis of pullulan were predicted and compared. The range standard for amino acid residue confidence pLDDT is as follows: pLDDT >90 means high confidence and highly reliable structure; PLDDT 70–90 means moderate confidence and relatively reliable structure; PLDDT of 50–70 means low confidence and less reliable structure; PLDDT <50 means very low confidence, and the accuracy of the structure may be poor. PTM is a score that measures the accuracy of the entire protein folding, and the higher the score, the better the predicted folding quality. The template modeling score for interface prediction, ipTM score of 0.8 or higher, indicates high prediction quality, with gray areas ranging from 0.6 to 0.8. However, cases below 0.6 are questionable. Meanwhile, the RMSD value can be used to measure the 3D structural similarity between two proteins, with smaller RMSD values indicating higher protein structural similarity.

### 2.5 Pan-genome and genome evolution

GET_HOMOLOGUES version 07112023 (Contreras-Moreira and Vinuesa, 2013) was used to search homologous genes of different *Aureobasidium* strains, including four reference species of *A*. *pullulans* with whole genome sequencing (Gostinčar et al., 2014). The single copy homologous genes of different strains were connected and combined respectively, then the protein sequence was compared with the MAFFT software (default parameters), the sequences were clipped with TrimAl, and the phylogenetic tree was constructed with MEGA v7.0.14 through the Neighbor-Joining (NJ) method. The Bootstrap value was set to 1,000.

### 2.6 Transcriptional factor analysis for crucial genes

The upstream promoters of the α-1,3-glucan synthase encoding gene (*AGS2*) and the associated genes were searched through BioEdit v7.0.9.0 and DNAMAN, and then predicted in the YESTRACT+ website (http://www.yeastract.com/) (Monteiro et al., 2020).

## 3 Results

### 3.1 Genome analysis of *A. melanogenum* TN3-1

**FIGURE 1:**
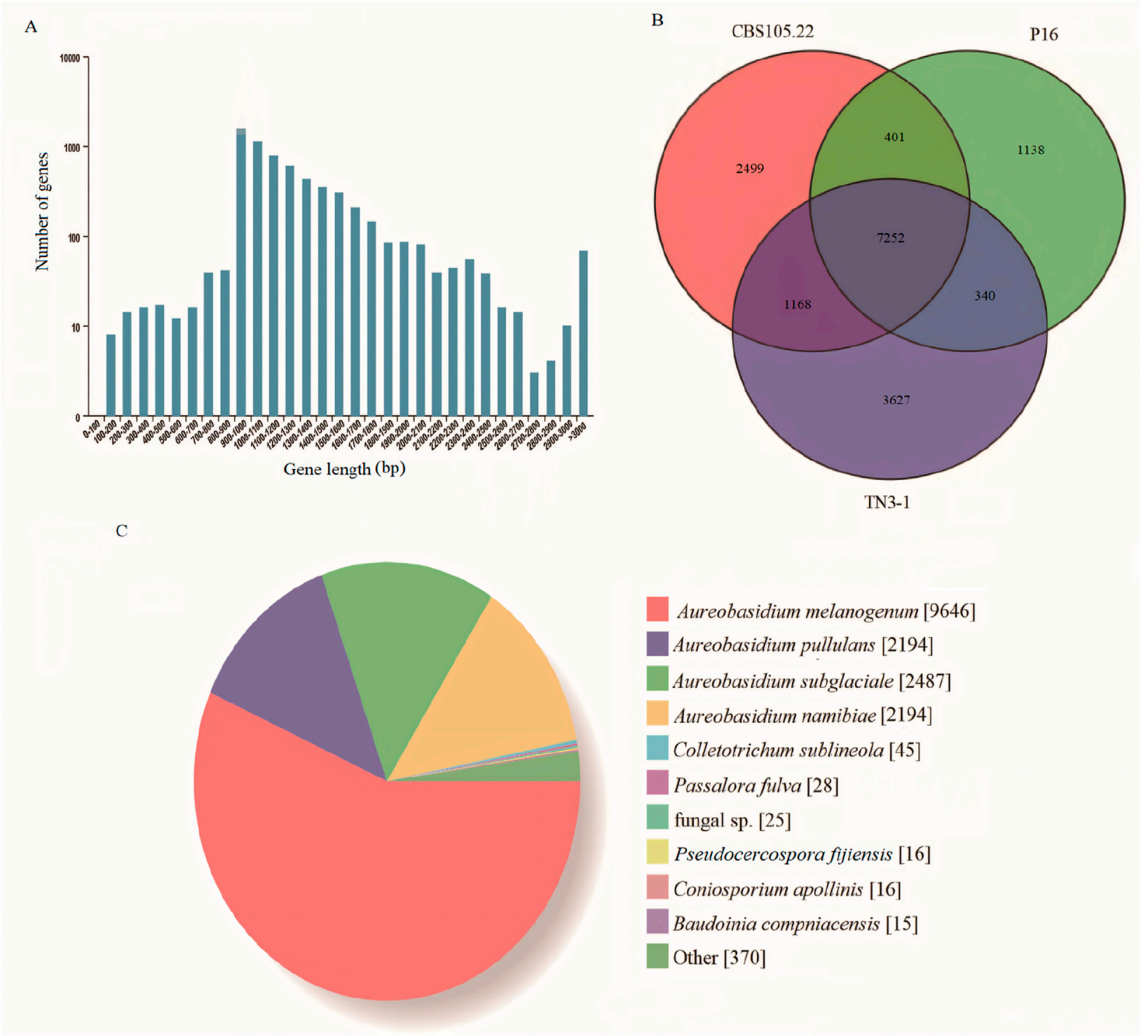
General genomic characteristics of the TN3-1 strain. **(A)** Gene distribution characteristics of TN3-1; **(B)** The common and unique genes of three reported *Aureobasidium* strains; **(C)** Distribution of homologous genes in the TN3-1 strain.

The downloaded genomes of *Aureobasidium* spp. are shown in Table 1, and the phenotypes of most strains have been validated (Chen L. et al., 2020; Chen TJ. et al., 2020; Chen et al., 2018; Zhang et al., 2018; Xue et al., 2019; Jia et al., 2023; Gostinčar et al., 2014). According to studies, the strain TN3-1 was collected from honey-comb (Xue et al., 2019). It can tolerate high osmotic pressure environment and produce pullulan, which is quite valuable for the research of microbial polyploidization (Chen L. et al., 2020). The results showed that the TN3-1 strain had a genome size of 51.6 Mb, GC content of 52.33%, and a total of 17,915 coding genes, most of which were 900–1,000 bp long with the average length of genes being 1,562 bp (Figure 1A) (Table 2). Meanwhile, TN3-1 strain has the highest number of specific genes, which may ensure its uniqueness (Figure 1B) (Table 2). Analysis of the specific genes showed that the specific genes of the high pullulan producing strain TN3-1 were 3,627, accounting for 28% of the whole genome (Figure 1B). Notably, both *A. melanogenum* TN3-1 and *A. melanogenum* CBS105.22 had occurred genome duplication, with almost every gene in the genome being duplicated (Jia et al., 2023). In addition, the homologous sequence comparison of all the protein sequences of TN3-1 strain in the NCBI-NR (Non-Redundant) database showed that, most of the genes are homologous genes with *A*. *melanogenum* (9,646) and *A. pullulans* (4,388), respectively. Quite a few genes also have high homology relationships with *Aureobasidium subglaciale* (2,487) (Figure 1C), indicating that this strain may be a completely different strain from *Aureobasidium* spp.

**TABLE 1:** Different fungal genomes download based on the available researches.

Strains	Accessions
*Aureobasidium pullulans* P25	GCA_003574545.1
*Aureobasidium melanogenum* P16	GCA_019915885.1
*Aureobasidium melanogenum* TN3-1	GCA_017949655.1
*Aureobasidium melanogenum* CBS105.22	GCA_018290055.1
*Aureobasidium melanogenum* P5	GCA_025604605.1
*Aureobasidium pullulans* var. namibiae CBS147.97	GCF_000721765.1
*Aureobasidium pullulans* var. subglaciale EXF-2481	GCF_000721755.1
*Aureobasidium pullulans* var. pullulans EXF-150	GCF_000721785.1
*Aureobasidium melanogenum* CBS 110374	GCF_000721775.1
*Aureobasidium* sp. P6	GCA_003992365.1
*Aureobasidium melanogenum* HN6.2	GCA_002156615.1
*Aureobasidium* sp. SLJ-2021a	GCA_019677095.1
*Aureobasidium zeae*	GCA_017580825.2
*Saccharomyces cerevisiae* S288C	GCF_000146045.2
*Schizosaccharomyces pombe* 972h-	GCF_000002945.1
*Yarrowia lipolytica* CLIB122	GCF_000002525.2
*Aspergillus niger*	GCF_000002855.4
*Blastomyces parvus* UAMH130	GCA_002572885.1
*Lepidopterella palustris* CBS459.81	GCA_001692735.1

**TABLE 2:** The general genomic characteristics of different *Aureobasidium* strains (BioProject: PRJNA661658).

Strains	Max length (bp)	N50 length (bp)	GC (%)	Genome size (bp)	Gene numbers	Gene length (bp)
P16	3,518,807	2,256,789	49.99	26,098,626	9,308	14,341,028
TN3-1	4,274,497	2,187,685	50.04	51,646,945	17,915	20,405,225
CBS105.22	4,273,107	2,075,361	49.99	48,644,395	17,817	27,348,966

Strains	Gene percentage (%)	Gene average length (bp)	% Of genome (internal)	Specific genes	NRPS	T1PKS
P16	54.95	1,524	45.05	1,138	1	2
TN3-1	39.51	1,562	60.49	3,627	3	4
CBS105.22	56.22	1,547	43.78	2,499	1	5

**FIGURE 2:**
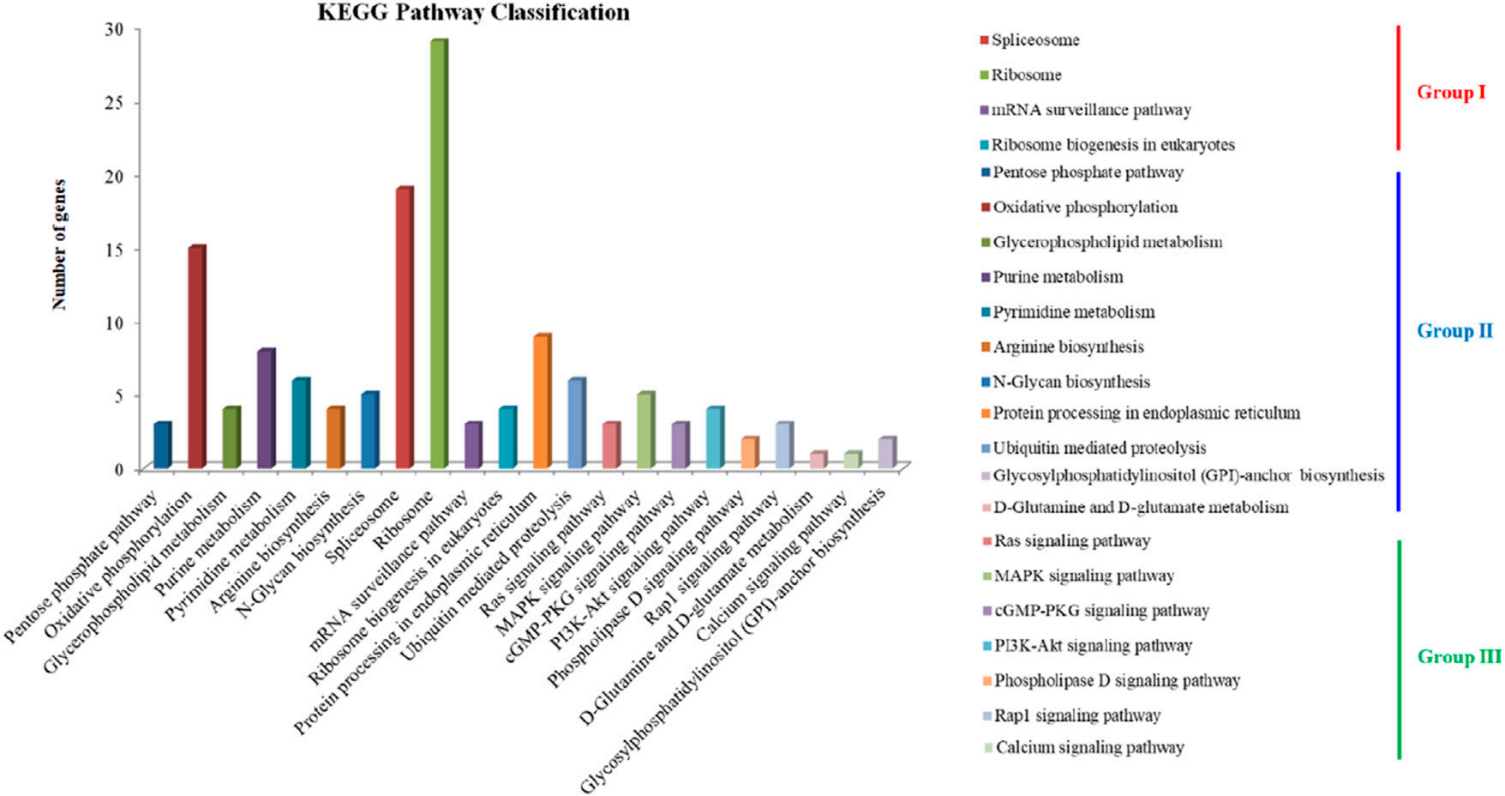
Enrichment analysis of the unique genes in the TN3-1 strain.

The secondary metabolites annotation showed that TN3-1 strain contained 3 NRPS metabolic gene clusters (MGCs) and 4 type I PKS gene clusters (Table 2). Further analysis showed that, the PKS MGCs may be involved in the synthesis of secondary metabolites such as elsinochrome A/B/C, melanin, and naphthopyrone. KEGG annotation of these specific genes showed that they were involved in ∼130 metabolic pathways, which could be divided into three groups: i) the processing of genetic information such as DNA repair and recombination, RNA transport and ribosome generation; ii) the metabolism related processes such as pentose phosphate pathway, respiratory chain oxidative phosphorylation, the glycosylphosphatidylinositol (GPI) biosynthesis and D-glutamine metabolism; iii) the metabolic pathway processes such as calcium ion signaling pathway, MAPK and RAS signaling pathway (Figure 2). The characteristics of specific genes in P16 strain was similar with that of TN3-1, but that in CBS105.22 strain did not show the Ras signaling pathway and the metabolic pathway related to the glycosylphosphatidylinositol (GPI) biosynthesis. The MAPK signaling pathway within the CBS105.22 strain was mainly associated with cell wall pressure response (K11244). However, in the pullulan producing strain P16 and TN3-1, the MAPK signaling pathway was mainly related to osmotic pressure response.

Interestingly, we found some crucial genes from the specific genes of TN3-1 strain, such as type II polyketo synthase-non-ribosomal peptide synthase, DNA repair protein, small GTP binding protein, 1,3-β-glucan transferase GEL2, 1,3-β-glucan transferase GEL3, the Ras family protein, Rho/Rac family GTPase activating protein-like protein, vacuole separation protein 55, mitogen-activated protein kinase, reverse transcriptase, β-glucosidase and cell wall mannoprotein hsp150, and they played important roles in secondary metabolite synthesis, DNA repair, spore formation and cell wall synthesis against osmotic pressure (Supplementary Table S1). In contrast, the specific genes in P16 strain included extracellular signal-regulated kinase, guanine exchange factor GEF, mitogen-activated protein kinase, WD repeat protein, and glucoyltransferase family proteins, which played an important role in signal transduction, transport, chromosome modification, spore formation, activation of Rho-GTPase activity and regulation of cell wall synthesis (Supplementary Table S1). Besides, we also found that there were many unknown functional domains encoding non-characterized gene family proteins, such as DUF1479 domain protein, DUF1793 domain protein, DUF218 domain protein and the DUF89 domain protein (Supplementary Table S1). Evolutionary analysis showed that members of the DUFs protein family might have many important functions (Mudgal et al., 2015). The DUF family member DUF1742 domain protein was also found in strain CBS105.22, and the other specific genes included α-1,2-mannosyltransferase mnn23, mitogen-activated protein kinase, stress response A/B domain protein, canavalin A-like lectin/glucanase, and copper Zinc superoxide dismutase like protein (Supplementary Table S1).

### 3.2 TN3-1 strain had a unique pullulan synthesis gene

**FIGURE 3:**
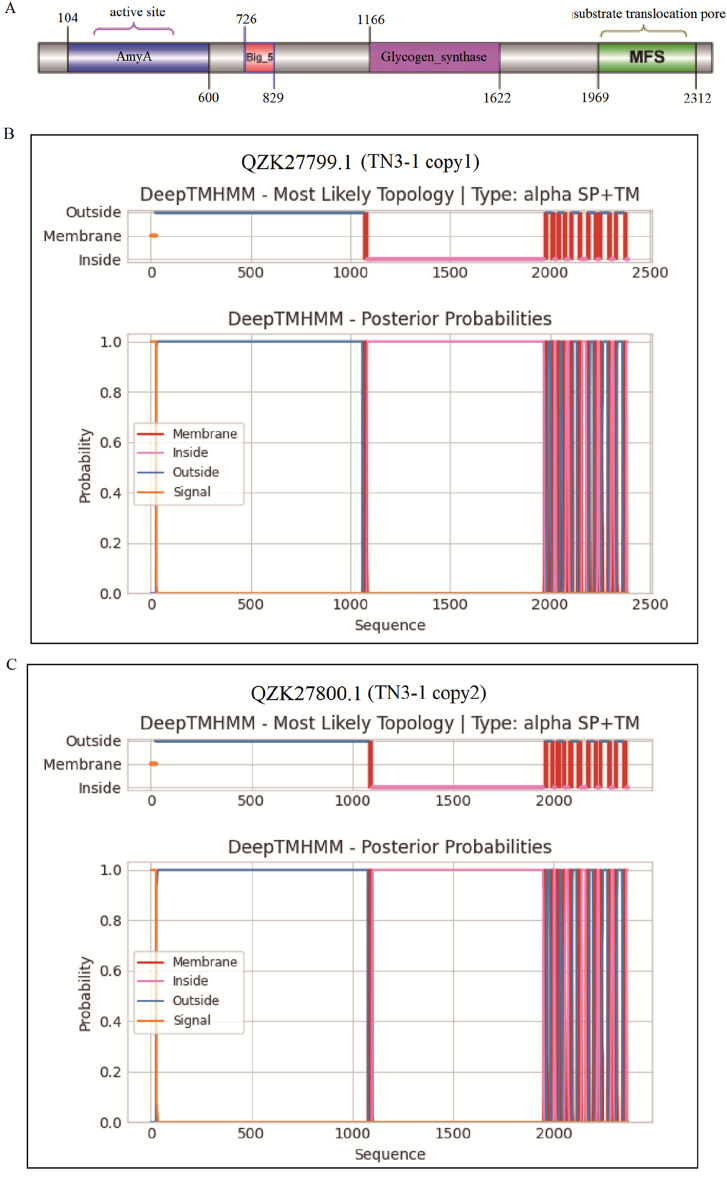
The conserved domain and transmembrane analysis of α-1,3-glucan synthase in TN3-1. **(A)** Domains of Ags2 in the TN3-1 strain; **(B)** Transmembranes of the copy1 Ags2 protein (QZK27799.1) in TN3-1; **(C)** Transmembranes of the copy2 Ags2 protein (QZK27800.1) in TN3-1.

The α-1,3-glucan synthase (Ags2) (EC 2.4.1.183) has been reported to play a crucial role in catalyzing the synthesis of pullulan, and this gene is prevalent in the strains of *Aureobasidium* (Chen TJ. et al., 2020; Chen et al., 2018). However, the α-1,3-glucan synthase coding gene was not found in the genomes of *Y. lipolytica* and *S. cerevisiae*, which might explain the reason why they could not produce pullulans. The accession of the two α-1,3-glucan synthase (Ags2) of TN3-1 strain was QZK27799.1 (copy1) and QZK27800.1 (copy2), respectively. Further analysis showed that α-1,3-glucan synthase had four conserved protein domains (Figure 3A), which might play a synergistic role in pululan synthesis (Chen TJ. et al., 2020; Jia et al., 2023; Qi et al., 2020). The core catalytic domain belonged to the alpha-amylase family, and alpha-1,3-glucan was also one of the components of the cell wall. We further analyzed the transmembrane structure of Ags2 proteins, and found that the two homologous Ags2 proteins of TN3-1 strain might be located on the cell membrane and were the membrane proteins. The transmembrane mode of two Ags2 proteins is similar (Figures 3B,C).

**FIGURE 4:**
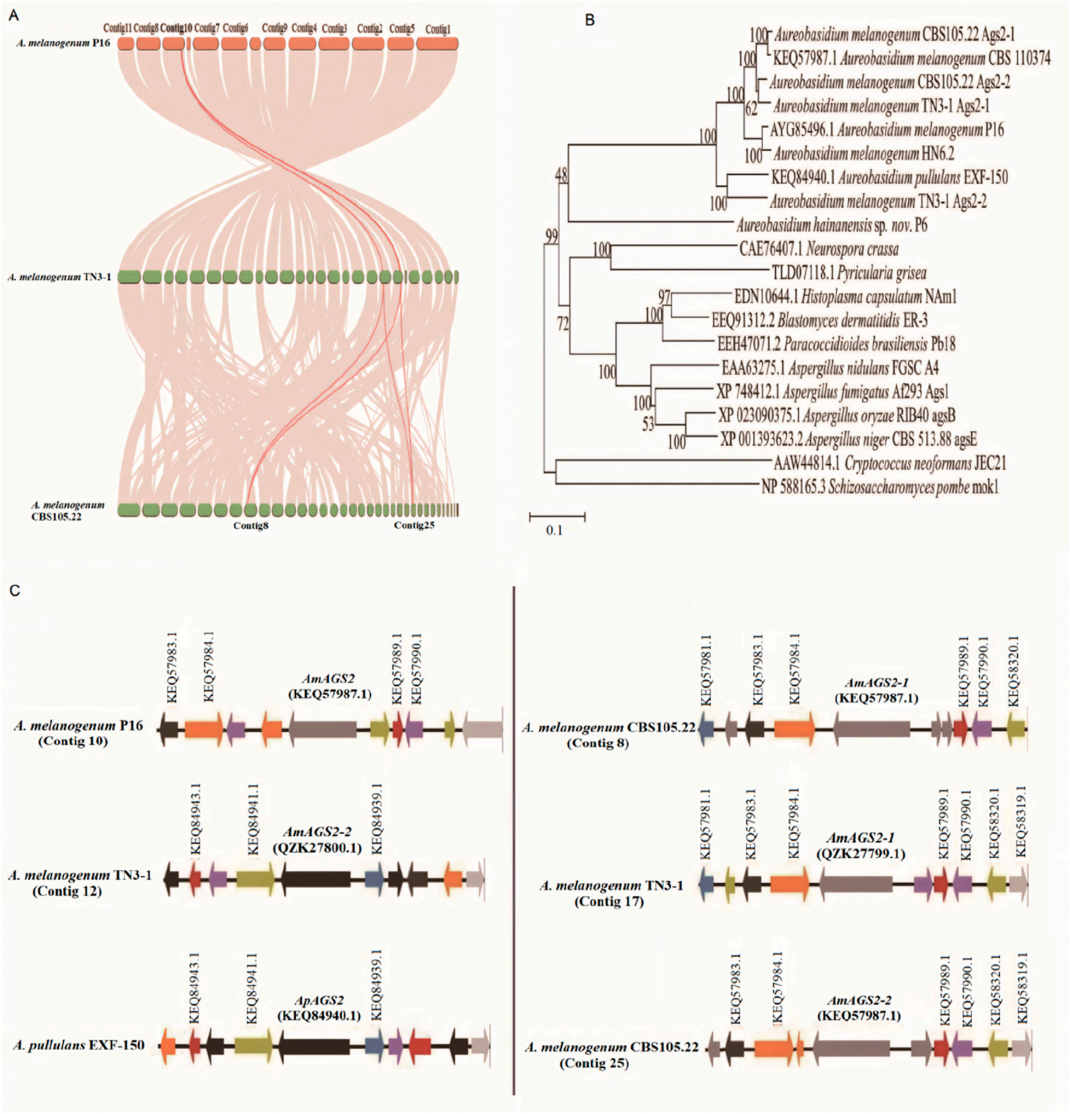
The α-1,3-glucan synthase encoding genes of different *Aureobasidium* strains. **(A)** The collinearity of *AGS2* genes; **(B)** The phylogenetic tree of Ags proteins; **(C)** Arrangement of the *AGS2* gene and its neighboring genes.

In genetics, *Ka/Ks* or *d*_*N*_ */d*_*S*_ represents the ratio between non-synonymous substitution (*Ka*) and synonymous substitution (*Ks*), and this ratio means whether there is selection pressure acting on this protein-coding gene. A nucleotide variation that does not result in an amino acid change is called a synonym mutation, and the reverse is called a non-synonym mutation. It is generally believed that synonymous mutations are not subject to natural selection, while non-synonymous mutations are subject to natural selection. If *d*_*N*_ */d*_*S*_ > 1, it is considered to have positive selection effect, if *d*_*N*_ */d*_*S*_ = 1, it is considered to have neutral selection, if *d*_*N*_ */d*_*S*_ < 1, it is considered to have purification selection (Álvarez-Carretero et al., 2023). The results showed that omega *(d*_*N*_ */d*_*S*_ *)* value was only 0.04, *d*_*N*_ */d*_*S*_ < 0.25, and the synonymous mutation rate was greater than the non-synonymous mutation rate. The results indicated that the *AGS2* gene of TN3-1 strain was purified and selected in the process of evolution, eliminating harmful mutations to maintain its important biological functions, and gradually retained for the offspring during evolution. Whole-genome collinearity analysis showed that the two *AGS2* genes of TN3-1 strain had collinearity with that of the P16 strain, while the two *AGS2* genes of CBS105.22 strain had collinearity with each *AGS2* gene of the TN3-1 strain (Figure 4A). Furthermore, the phylogenetic tree results showed that one copy of the *AGS2* gene (QZK27799.1) in TN3-1 strain was genetically related to the two copies of CBS105.22 and the *AGS2* gene from P16, while the other *AGS2* gene (QZK27800.1) of TN3-1 had relationships with *A. pullulans* EXF-150 (Figure 4B). Thus, it can be inferred that the three strains may have a common ancestor, the genome duplication events may occur before species differentiation, and most homologous genes have the same or similar functions, similar regulatory pathways, and conserved functions. Furthermore, it was found that the two *AGS2* genes of strain CBS105.22 had high similarity values, and all of them were close to one *AGS2* gene of strain TN3-1. The duplicated *AGS2* genes of strain CBS105.22 may be parologous genes produced by genome duplication. Due to the lack of original natural selection force, the duplicated *AGS2* gene can be freely mutated and may acquire new functions according to the theory of genome polyploidization (Albertin and Marullo, 2012). The collinearity analysis of the *AGS2* gene showed that the *AGS2* gene was located in the collinearity block of the TN3-1, P16 and CBS105.22 strains, and had obvious paralogous characteristics in CBS105.22 strain. Further analysis showed that one copy (QZK27799.1) of TN3-1 had an obvious collinearity relationship with the arrangement of the two *AGS2* genes in strain CBS105.22, and the similarity value of this gene in CBS105.22 was high (96%). In addition, the gene arrangement of the other *AGS2* gene (QZK27800.1) in TN3-1 strain was more consistent with that of *A. pullulans* EXF-150 than the other two strains (Figure 4C), and the similarity between this gene was about 85%, higher than that of the P16 strain (81%) (Supplementary Table S2). That meant that this *AGS2* gene might have associations with *A. pullulans* strain, and underwent the process of natural selection, gradually being inherited by eliminating harmful mutations.

**FIGURE 5:**
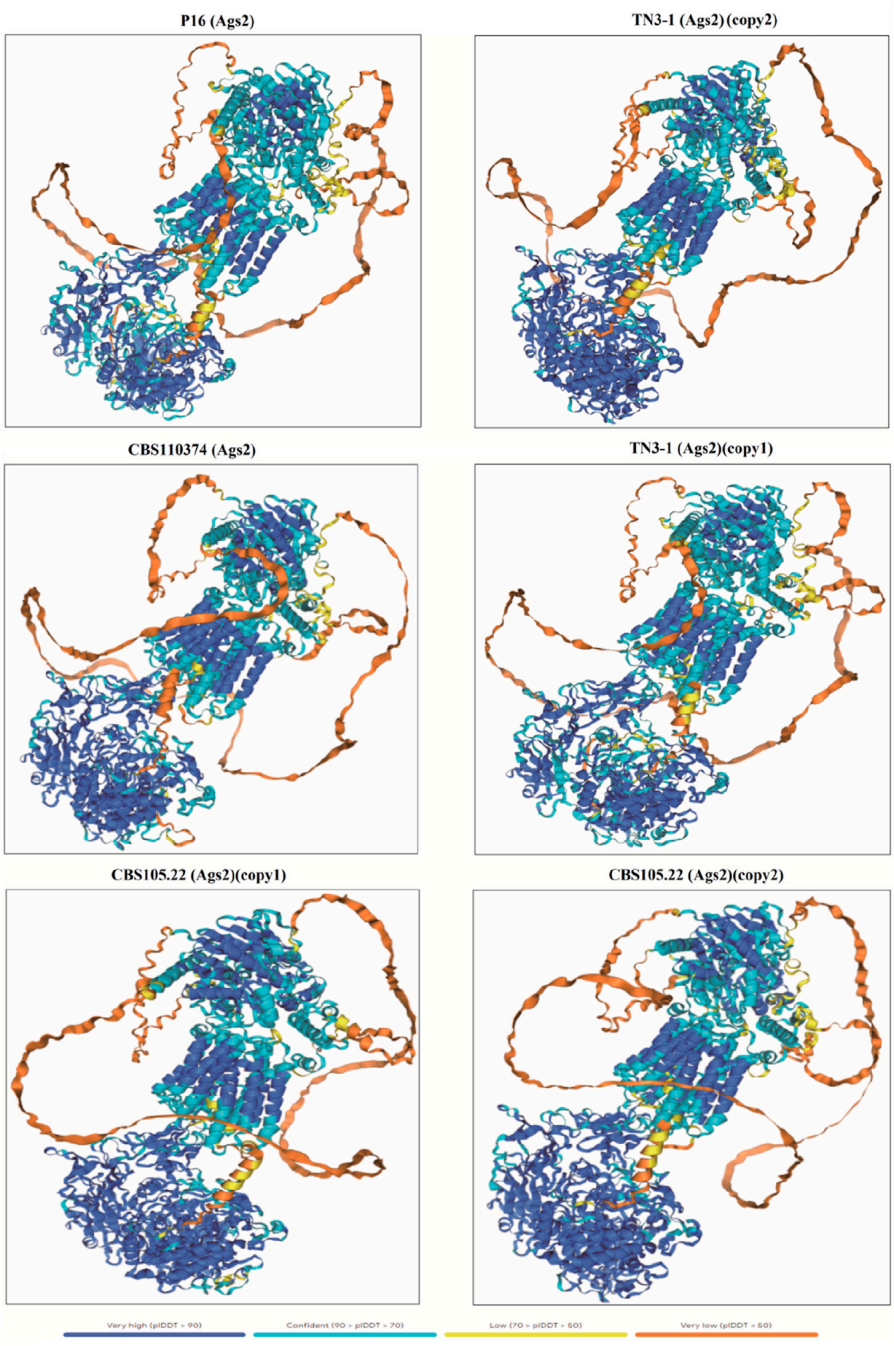
The 3D structure comparison of α-1,3-glucan synthase from different strains of *Aureobasidium melanogenum* spp.

**FIGURE 6:**
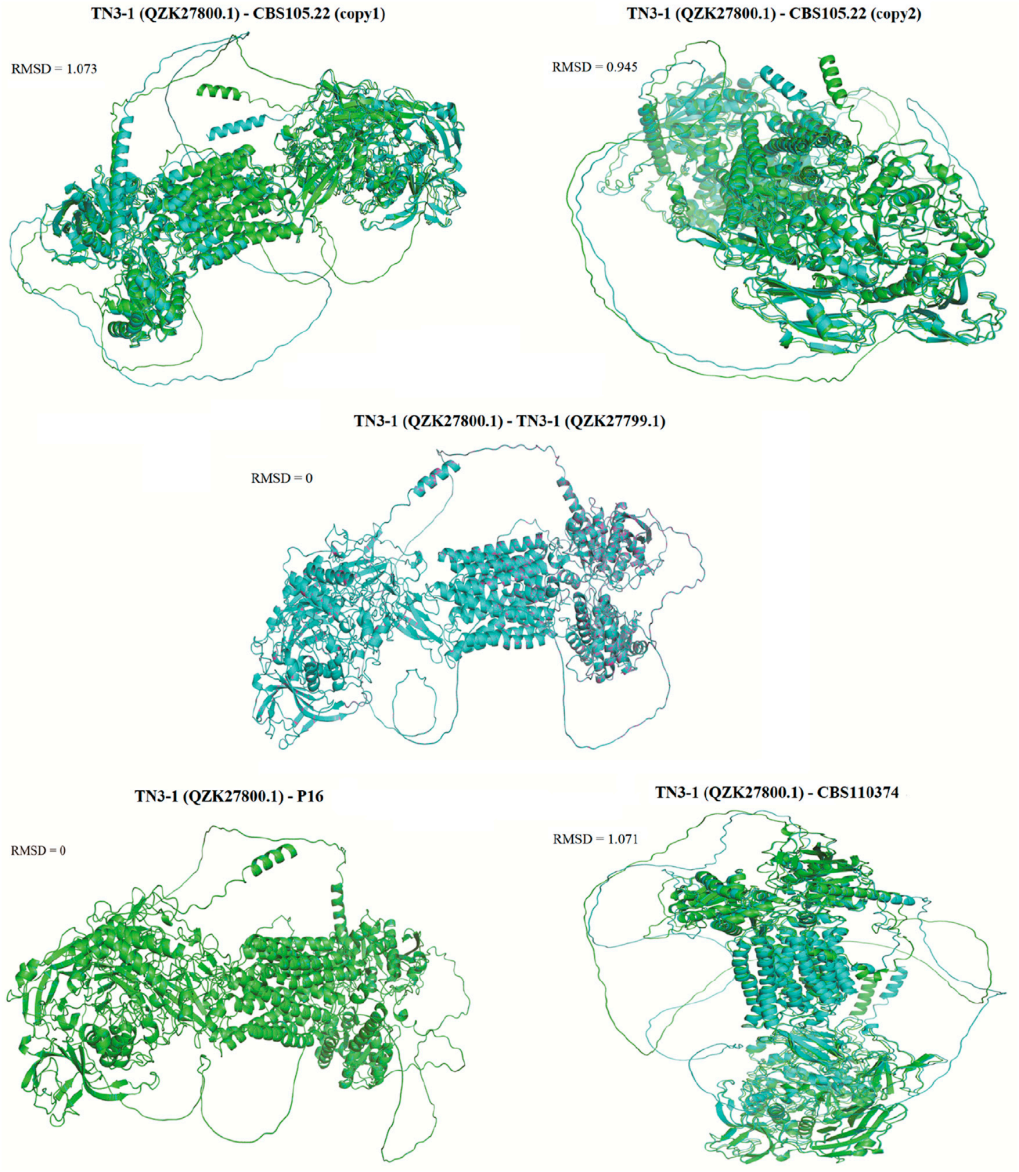
Comparison of the 3D structural similarity between the core Ags2 (QZK2780.1) of TN3-1 and the other Ags2 proteins. The diagram showed the protein structure coincidence degree, and the higher the degree of protein overlap, the smaller the RMSD value.

Furthermore, a 3D model of the α-1,3 glucan synthase was also constructed to further predict its 3D structure. The results showed that the 3D structure of the crucial pullulan synthesis enzyme (QZK27800.1) in TN3-1 strain was different from *A*. *melanogenum* CBS105.22 and *A*. *melanogenum* CBS110374, but it was identical to that of P16 strain (Figures 5, 6). However, the 3D structure of the other α-1,3-glucan synthase (QZK27799.1) in TN3-1 strain was also identical to the crucial pullulan synthesis enzyme (QZK27800.1), but the sequence similarity of them was not so high (Supplementary Table S2) (Figures 5, 6). This means that the correct folding pattern of Ags2 might have associations with pullulan production, but it is possibly not the decisive factor for pullulan production.

### 3.3 TN3-1 strain activated distinct pathways in response to environmental changes

Under low nitrogen and osmotic stress environments, yeast can respond to osmotic stress through two signaling pathways, namely, the MAPK cascade cell wall integrity signaling pathway and Ras/cAMP signaling pathway, which helps cells search for nutrients (Vinod et al., 2008). Multiple binding sites of transcription factor (TF) Tec1 and Ste12 were found in the β-1,3-glucan *(FKS)* gene promoter of the TN3-1, P16 and CBS105.22 strains. The two transcription factors can synergically regulate mycelia growth in different metabolic pathways. The transcription factor Swi4/6 was a zip-specific transcriptional activator in regulating the cell wall integrity signaling pathway (CWI), however, there was no TF binding sites of Swi4/6 in TN3-1 and P16 strains, but the TF binding site (CACGAAA) was only found in the promoter of the β-1, 3-glucan synthase encoding gene of CBS105.22 strain (Table 3). In the cell wall integrity pathway, *Bck2* gene first activates the expression of M/G1 gene Swi4 by combining with transcription factor Mcm1, and then further regulates the expression of downstream genes by transcription factor Swi4 (Bastajian et al., 2013). In strain CBS105.22, we found multiple binding sites (CCYWWWNNRG) of Mcm1 in the *SWI4* promoter region, indicating a complex regulation network. In addition, in TN3-1 and P16 strains, the binding sites (ACCAGC) of transcription factor Ace2 were observed in the upstream region of the *AGS2* gene (Table 3). The C-terminal structure of ACCAGC was identical to that of transcription factor Msn2, which played an important role in regulating mycelium morphology, adhesion, membrane formation and cell separation (Yang et al., 2020). Thus, due to the differences in gene regulation, the expression of *AGS2* gene may be relatively weak in the non-pullulan producing strain CBS105.22, while the CWI pathway may play a dominant role in CBS105.22 strain, so as to cope with the osmotic stress response.

**TABLE 3:** Comparison of transcription factor binding sites in different promoter regions.

Promoter	Strains	SWI4/6	Promoter	Strains	MSN2
*FKS*	P16	-	*PFK*	P16	-
	TN3-1	-		TN3-1	-
	CBS105.22	+		CBS105.22	+

Promoter	Strains	ACE2	Promoter	Strains	MSN2
*AGS2*	P16	+	*PGM*	P16	+
	TN3-1	+		TN3-1	+
	CBS105.22	-		CBS105.22	-

+: at least one transcription factor binding site was presented.

-: no transcription factor binding sites were detected.

Studies have shown that cells produce more ATP for the metabolism of polymeric precursors to synthesize more pululans under low nitrogen conditions, and the expression of glycolysis genes is mainly regulated by transcription factor Msn2 (Rødkær and Færgeman, 2014; Gorner et al., 2002). However, we found no Msn2 binding site (AGGGG) in the upstream region of phosphofructokinase gene (*PFK*) in TN3-1 and P16 strains, while in strain CBS105.22, the Msn2 binding site (AGGGG) existed in the upstream of the two *PFK* gene copies (Table 3). Therefore, in the pullulan producing strain P16 and TN3-1, carbon flow was possibly more concentrated in the glycogen synthesis pathway, while in strain CBS105.22, the carbon flow might go through the glycolysis pathway to generate acetyl CoA, which was conducive to growth (Kuang et al., 2018). In addition to the *AGS2* gene, other important genes involved in the synthesis process of pullulan include the UDPG-pyrophosphorylase gene (*UGP*), the glucose-mutase phosphate gene (*PGM*), the UDPG-glucosyl transferase gene (*UGT1*), and the glucokinase gene (*Gluk*). Among them, *PGM* catalyzes the mutation of glucose 6-phosphate to form glucose 1-phosphate, *UGP* catalyzes glucose 1-phosphate and UTP to form UDPG, and *UGT1* catalyzes the substrate UDPG to synthesize polysaccharide chain, which are considered to be important rate-controlling steps in the synthesis of pullulan (Li et al., 2015). Through the transcription regulation analysis, the results showed that in TN3-1 and P16 strains, there was an Msn2 transcription factor regulatory site (AGGGG) in the promoter region of the *PGM* gene (Table 3). Under the regulation of transcription factors, the high expression of this gene can produce a large amount of glucose-1-phosphate, which leads to the process of glycogen synthesis*.* In addition, the binding site (CCCCT) of Msn2 was also found in *Y. lipolytica* and *S. cerevisiae*, however, it was not observed in strain CBS105.22. The expression of this gene may be relatively weak during the synthesis of pullulan precursors, so the carbon flow possibly diverted to cell growth in this strain.

### 3.4 Efficient energy supply provides strong assurance

**FIGURE 7:**
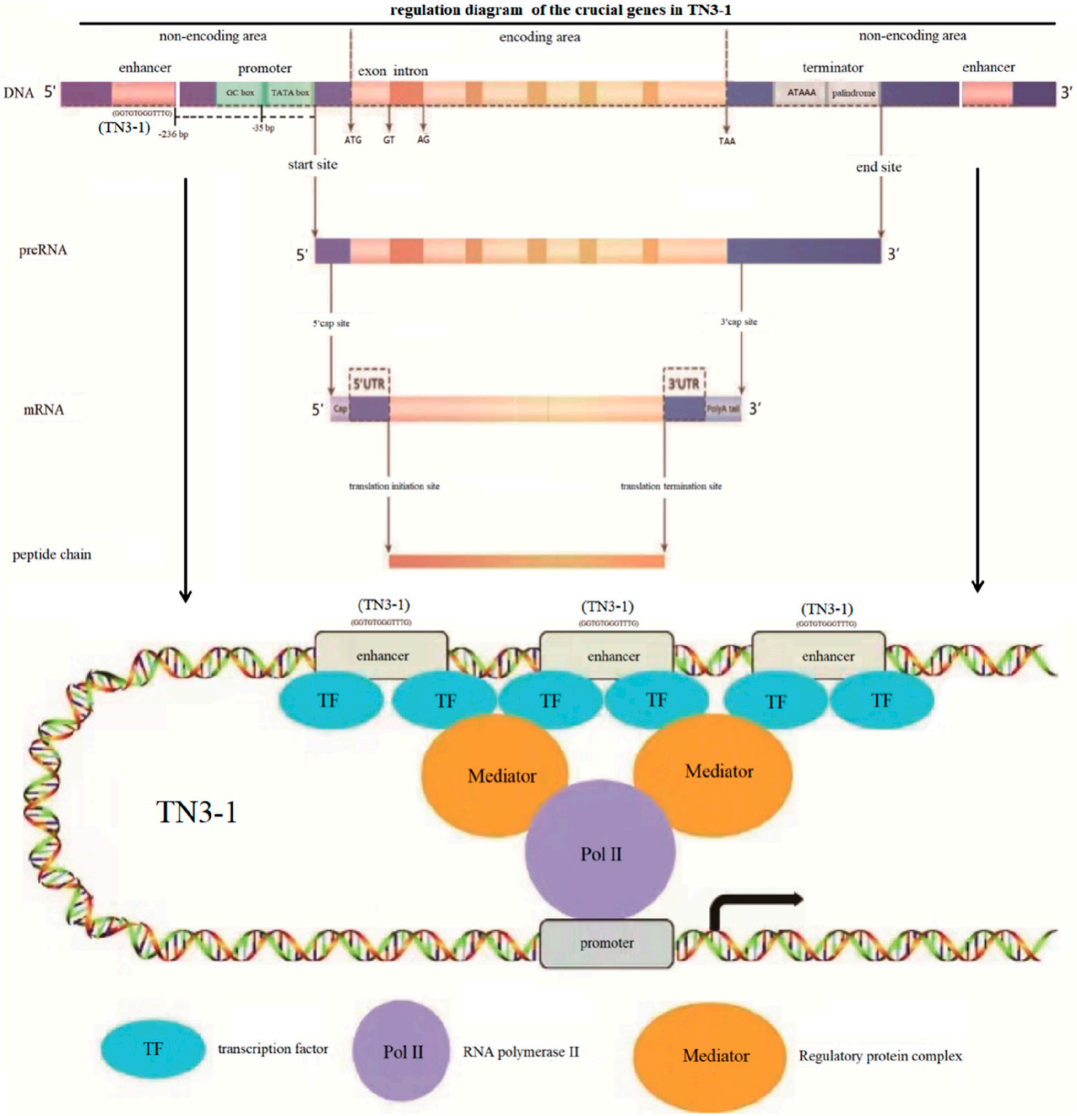
Schematic diagram of upstream promoters and regulation of the crucial genes in TN3-1.

Analysis of the crucial genes in TCA cycle and respiratory chain showed that in the TN3-1 strain, one copy of the succinyl-CoA synthetase β subunit encoding gene contained an enhancer at the distance of −236 bp from the transcription start site. The core sequence of this enhancer was “GGTGTGGGTTTG”, while no enhancer was found in the promoter region of the P16 and CBS105.22 strains. Meanwhile, a GC-box (GGGCGG) was also observed at −35 bp from the transcription start site, which could enhance the transcription start frequency (Figure 7). The above characteristics may ensure the efficient energy supply of TN3-1 strain in the process of pullulan synthesis.

## 4 Discussion

Consistent with existing research (Jia et al., 2023), we found that TN3-1 strain had a larger genome and more genes than any other reported or available strains of *Aureobasidium* spp. Especially, it carried many more specific genes than the other reported *Aureobasidium* strains. The specific genes of TN3-1 strain involved the osmotic pressure related genes, as well as the genes of wall synthesis, etc. These specific genes may have different characteristics in osmotic stress response, spore formation or free radical scavenging. According to the gene collinearity, the specific genes in TN3-1, P16 and CBS105.22 strains may originate from a common ancestor and have different functions with differentiation. Among the specific genes, several Ras and GTP protein family members as well as the genes related to regulation of cell wall synthesis were found in TN3-1 strain, which might have special advantages in coping with osmotic stress response. In addition, there are a certain number of homologous genes with different species of *Aureobasidium* spp., indicating that TN3-1 strain is possibly a heterozygous strain. Indeed, it was speculated to be a new species formed by the fusion of different *Aureobasidium* strains (Jia et al., 2023). In this study, we proposed that the TN3-1 strain carried a unique *AGS2* gene. However, it seemed that the unique *AGS2* gene of TN3-1 did not underwent positive selection, but retained its original functions. Interestingly, studies showed that only one of the two homologous α-1,3-glucan synthase coding genes played a crucial role in TN3-1 strain, while the other *AGS2* gene could not catalyze the synthesis of pullulan. The two *AGS2* genes of TN3-1 were not directly the “superimposed” relationships (Chen L. et al., 2020; Jia et al., 2023; Qi et al., 2020), which meant that gene dose effect might not be applicable to *Aureobasidium* spp. with genome duplication. This may be related to the ancient evolutionary status of the TN3-1 strain (late Oligocene) (Jia et al., 2023), which is more ancient than other strains of *Aureobasidium* spp. After genome duplication, one copy of the *AGS2* gene might lose its original function due to the accumulation of harmful mutations, and the subsequent evolution of the non-pullulan producing strain CBS105.22 inherited one copy of the *AGS2* gene (QZK27799.1) from TN3-1 strain, and further underwent gene duplication. After all, in the genomes of eubacteria and archaea, ∼30%–50% of genes belonged to paralogues, which were higher in eukaryotic genome (Koonin, 2005). Previous studies speculated that TN3-1 strain was formed by the fusion of the ancestor of CBS105.22 and an ancient strain similar to P16 strain, thus possessing the characteristics of both strains (Jia et al., 2023). In this case, TN3-1 can tolerate high temperature environments, which is similar to the CBS105.22 strain isolated from granuloma of child, and also has the ability of pullulan production similar to the P16 strain.

In this article, we found that the TN3-1 strain may respond to environmental changes by initiating specific signaling pathways. The response to osmotic stress is demonstrated through the cell wall integrity signaling pathway and the Ras/cAMP signaling pathway, which have been shown to be associated with the pullulans synthesis. For example, there are transcription factor binding sites in the upstream of some crucial genes, while these regulatory sites do not exist in P16 and CBS105.22 strains. Similarly, regulatory sites of transcription factors were also found in the upstream of the glycolytic genes. Under the regulation of transcription factors, high expression of these genes can synthesize a large amount of glucose-1-phosphate, leading to a shift in metabolic flow towards glycogen synthesis. However, in the CBS105.22 strain, the expression of these genes may be relatively weak, resulting in a greater shift in carbon flow towards cell growth. This could be supported by the previous studies (Chen et al., 2018; Xue et al., 2019). Combined with the above analysis, it was speculated that under osmotic pressure conditions, different strains of *Aureobasidium* spp. could activate different signaling pathway response strategies in response to environmental changes, which was in line with the characteristics of adaptive evolution (Causton et al., 2001). The pullulan producing strain TN3-1 and P16 may be mainly regulated by the Ras/cAMP signaling pathway, while strain CBS105.22 may resist osmotic pressure mainly through the cell wall integrity signaling pathway (CWI) due to the high sensitivity and permeability of biofilm to glucose concentration. In strain CBS105.22, the *FKS* gene was regulated by transcription factor Swi4 to synthesize β-1,3-glucan for cell wall synthesis, which showed filamentous characteristics (Hochstenbach et al., 1998). As reported, previous studies showed that enlarged cells and thick-walled spores were the main causes of pululan formation, while conidium and mycelium had no effect on the synthesis of pullulan (Campbell et al., 2004). Thus, the Ras/cAMP signaling pathway may be a major pathway for pullulan synthesis under low nitrogen and osmotic stress conditions, and the downstream gene expression is mainly regulated by transcription factor *ACE2*, *MSN2* and other TFs (Bastajian et al., 2013; Estruch, 2000; Kottom and Limper, 2013). In addition, the enhancer in the upstream of the TCA cycle associated gene may enhance the expression of this gene, ensuring efficient energy supply during metabolic process. Usually, the transcriptional enhancer has a transcriptional enhancement effect, which can generally increase the transcription frequency by 10–200 times (Haberle and Stark, 2018). In the process of pullulan synthesis, GTP synthesis by succinyl-CoA synthetase in the TCA cycle is the only substrate level phosphorylation reaction, and the generated GTP can be used for energy supply or rapidly activate the Ras/cAMP signaling pathway, making it at an advantage in stress response.

Although we have further explored the mechanisms of stress tolerance and pullulan production, there still exist some shortcomings. First, we only supplemented the results based on previous studies and did not compare it with more polyploid strains, which limited the research scope. Second, our research was limited to the downloaded genomic data and lacked necessary experimental validations. Finally, we did not make analysis of the interactions between promoters and the crucial proteins through AlphaFold Server 3.0. Until now, NCBI has totally released 176 *Aureobasidium* genomes (3 July 2024) (https://www.ncbi.nlm.nih.gov/assembly/?term=Aureobasidium). With the rapid development of the next-generation sequencing (NGS) technology, more and more *Aureobasidium* genomes will be released. Full-scale genome analysis will be beneficial for discovering interesting strains and functional genes, revealing the potential molecular mechanisms of stress tolerance and pullulan synthesis.

## 5 Conclusion

On basis of previous studies, we further analyzed the genomic characteristics of TN3-1 strain and the possible molecular mechanisms of its high-yield ability of pullulan production. The genome duplication, gene function differentiation and promoter region changes of TN3-1 strain may be the important factors for its special phenotype. Meanwhile, it may have heterozygous advantages during the evolutionary process. However, the 3D structure of Ags2 protein may not be a key influencing factor in the synthesis of pullulans. It will be a type strain for studying of microorganisms synthesizing pullulans, and provide new insights for studying adaptive evolution of genome duplication in *Aureobasidium* spp.

## Data Availability

The raw data supporting the conclusions of this article will be made available by the authors, without undue reservation.
