# Supplementary figures and images for: Genomic analysis and mechanisms exploration of a stress tolerance and high-yield pullulan producing strain
Source: Front Genet. 2024 Sep 20;15:1469600. doi: 10.3389/fgene.2024.1469600 (PMC11449735; doi:10.3389/fgene.2024.1469600)

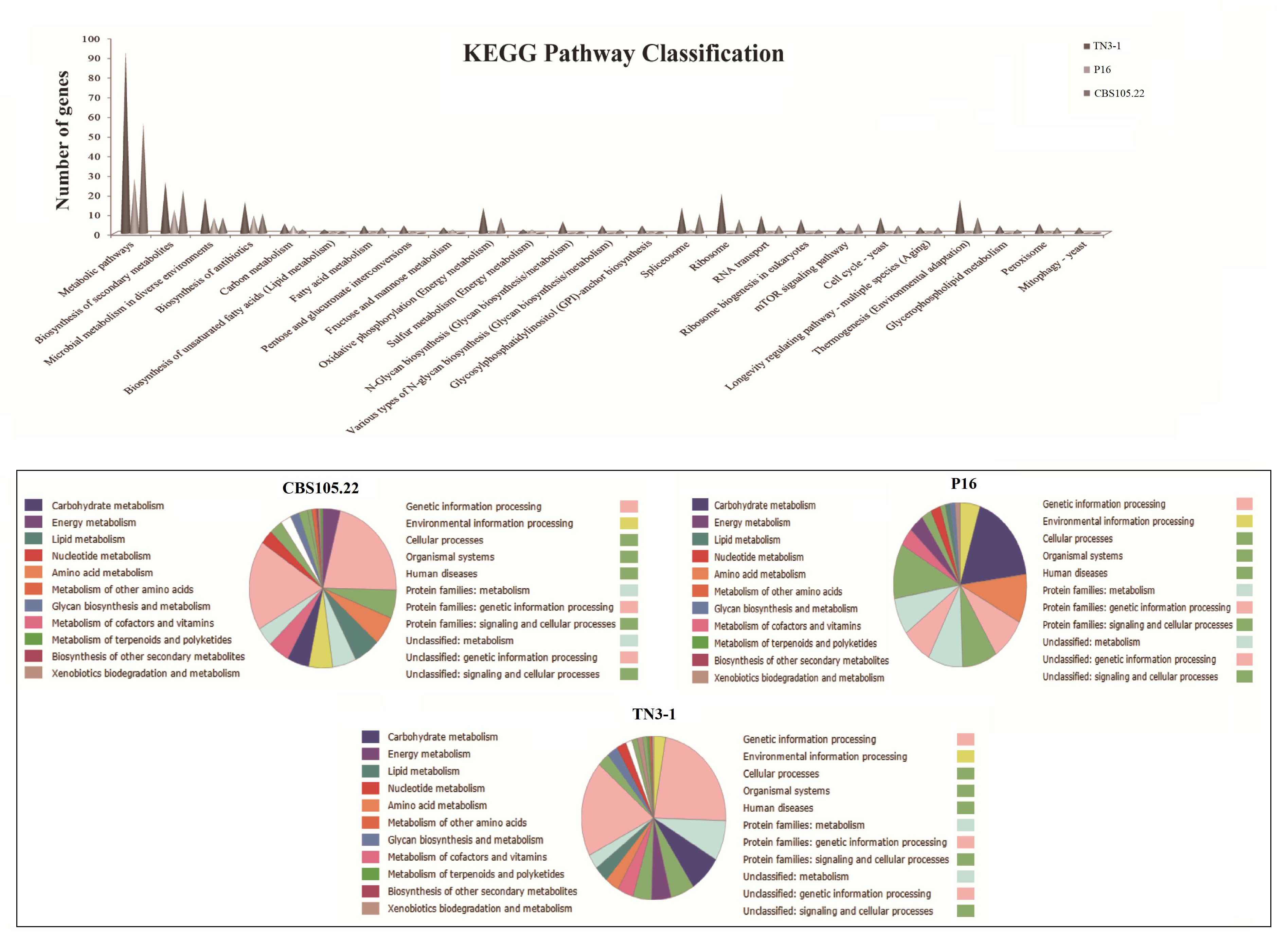

Supplement: Supplementary file 2 [file Image1.PNG]
